# Transcatheter Aortic Valve Implantation: Long-Term Outcomes and Durability

**DOI:** 10.14797/mdcvj.1201

**Published:** 2023-05-16

**Authors:** Parth V. Desai, Sachin S. Goel, Neal S. Kleiman, Michael J. Reardon

**Affiliations:** 1 Loyola University Medical Center, Maywood, Illinois, US; 2 Houston Methodist DeBakey Heart & Vascular Center, Houston Methodist Hospital, Houston, Texas, US

**Keywords:** transcatheter aortic valve implantation, long-term outcomes, durability, structural valve degeneration, severe aortic valve stenosis, surgical aortic valve replacement

## Abstract

Transcatheter aortic valve implantation (TAVI) has become the standard of care in symptomatic older patients with severe aortic stenosis regardless of surgical risk. With the development of newer generation transcatheter bioprostheses, improved delivery systems, better preprocedure planning with imaging guidance, increased operator experience, shorter hospital length of stay, and low short- and mid-term complication rates, TAVI is gaining popularity among younger patients at low or intermediate surgical risk. Long-term outcomes and durability of transcatheter heart valves have become substantially important for this younger population due to their longer life expectancy. The lack of standardized definitions of bioprosthetic valve dysfunction and disagreement about how to account for the competing risks made comparison of transcatheter heart valves with surgical bioprostheses challenging until recently. In this review, the authors discuss the mid- to long-term (≥ 5 years) clinical outcomes observed in the landmark TAVI trials and analyze the available long-term durability data emphasizing the importance of using standardized definitions of bioprosthetic valve dysfunction.

## Introduction

After nearly two decades of experience, transcatheter aortic valve implantation (TAVI) has altered the treatment landscape for severe aortic stenosis. Initially studied in inoperable and high-risk patients, TAVI was recently studied and found beneficial in patients with low surgical risk, leading to approval across all surgical risk profiles.^1,2^ Growing experience, advent of newer iterations of transcatheter heart valves, improved preprocedure planning, and smaller access sheaths have significantly minimized the periprocedural TAVI complications. Based on data from randomized controlled trials (RCTs), the latest professional society guideline^3^ has endorsed TAVI as an alternative to surgical aortic valve replacement (SAVR) for patients between 65 and 80 years (class 1), with a strong emphasis on shared decision-making between patients and the heart team with regard to lifetime management and the possible need for reintervention. TAVI in younger and lower-risk patients with otherwise longer life expectancies has appropriately raised concerns about transcatheter heart valve longevity and its effects on future reinterventions. In this review, we discuss mid- to long-term clinical outcomes and long-term durability data of TAVI from landmark clinical trials compared to surgical bioprostheses and its foreseeable implications.

## Mid-term Outcomes of TAVI

Short-, mid-, and long-term durations are arbitrarily defined in various TAVI studies. For this review, 5-year or longer follow-up data will be discussed to assess the mid-term or long-term outcomes of TAVI. The results of pivotal trials stratified by the Society of Thoracic Surgeons-Predicted Risk of Mortality (STS-PROM)^4^ are summarized in Tables 1 and 2. Patients are categorized as low, intermediate, or high risk based on Society of Thoracic Surgeons predicted risk of mortality at 30 days (following SAVR) of < 4%, 4% to 8%, or > 8%, respectively.^4,5^ Patients with a > 50% preoperative risk of mortality and morbidity at 1 year are considered to be inoperable or at extreme risk.^4,5^

**Table 1:** Long-term clinical outcomes of landmark TAVI trials in prohibitive and high-surgical-risk patients.


SURGICAL RISK	TRIAL	YEAR	INTERVENTION	TAVI VALVE TYPE	AGE (Y)	% MALE	FOLLOW-UP AT 5 YEARS	LONG-TERM OUTCOMES

Prohibitive risk	PARTNER 1B^8^ (Mean STS 11.7%)	2015	N = 358 179 TAVI 179 Standard therapy^†^	BE first-generation SAPIEN (Edwards)	83	46%	49/179 TAVI survived 5/179 SAVR survived	At 5 years, TAVI vs standard therapy: • All-cause death (71.8% vs 93.6%, ss) • CV mortality (57.5% vs 85.9%, ss) • Rehospitalization (47.6% vs 87.3%, ss) • All stroke (16.0% vs 18.2%, ss)

	CoreValve US Pivotal^9^ (Mean STS 10.4%)	2021	N = 639 639 TAVI compared with meta-analysisdata	SE First-generation CoreValve (Medtronic)	83	48%	158/639 survived at 5 years	At 5 years, TAVI: • All-cause death or major stroke (72.6%) • Major stroke (11.5%) • Major/life-threatening bleed (29.4%) • AV Reintervention (2.9%) • PPI (46.8%) • Aortic valve rehospitalization (48.3%) • Moderate or severe PVR (3.1%)

High risk	PARTNER 1A^10^ (Mean STS 11.7%)	2015	N = 699 348 TAVI 351 SAVR	BE first-generation SAPIEN (Edwards)	84	58%	Median follow-up 3.14 years (IQR 0·68–4·92)	At 5 years, TAVI vs SAVR: • All-cause death (67.8% vs 62.4%, ns) • Rehospitalization (42.3% vs 34.2%, ns) • All-strokes (15.9% vs 14.7%, ns) • Moderate or Severe PVR (TAVI > SAVR, ss)

	CoreValve US Pivotal^11^ (Mean STS 7.4%)	2018	N = 797 391 TAVI 359 SAVR	SE First-generation CoreValve (Medtronic)	83	53%	Median follow-up 49.9 months (TAVI), 41 months (SAVR)	At 5 years, TAVI vs SAVR: • All-cause death (55.3% vs 55.4%, ns) • Major stroke (17.5% vs 21.0%, ns) • Myocardial infarction (3.1% vs 3.3%, ns) • AV Reintervention (3.0% vs 1.1%, ss) • PPI (38.6% vs 22.3%, ss) • Moderate SVD (9.2% vs 26.6%, ss)


STS: Society of Thoracic Surgeons; BE: balloon-expandable; SE: self-expandable; TAVI: transcatheter aortic valve replacement; SAVR: surgical aortic valve replacement; CV: cardiovascular; PPI: permanent pacemaker implantation; PVR: paravalvular regurgitation; SVD: structural valve degeneration; AV: aortic valve; ss: statistically significant; ns: statistically nonsignificant; IQR: interquartile range ^†^ 140 (79%) of 179 patients in the standard treatment group underwent balloon aortic valvuloplasty.

**Table 2:** Long-term clinical outcomes of landmark TAVI trials in intermediate and low-surgical risk patients.


SURGICAL RISK	TRIAL	YEAR	INTERVENTION	TAVI VALVE TYPE	AGE (Y)	% MALE	FOLLOW UP%	LONG-TERM OUTCOMES

Intermediate risk	PARTNER 2^12^ (Mean STS 5.8%)	2020	N = 2032 1011 TAVI 1021 SAVR	BE second-generation SAPIEN XT (Edwards)	82	55	91% TAVI 81.4% SAVR	At 5 years, TAVI vs SAVR: • All-cause death or disabling stroke (47.9% vs 43.4%, ns) • All-cause death (46% vs 42.1%, ns) • Disabling Stroke (9.8% vs 8.6%, ns) • Rehospitalization (33.3% vs 25.2%, ss) • New PPI (15.5% vs 13%, ns) • AV Reintervention (3.2% vs 0.8%, ss) • Moderate to severe PVR (4.1% vs 0.42%, ss)

	SURTAVI^13^ (Mean STS 4.5%)	2022	N = 1660 864 TAVI 796 SAVR	SE CoreValve (84%) Evolut R (16%) (Medtronic)	80	56	93.7% TAVI 95.5% SAVR	At 5 years, TAVI vs SAVR: • All-cause death or disabling stroke (31.3% vs 30.8%, ns) • All-cause death (30.0% vs 28.7%, ns) • Valve thrombosis (0.5% vs 0.4%, ns) • AV Reintervention (3.5% vs 1.9%, ss) • Moderate or severe PVR (3.0% vs 0.7%, ss)

Low risk	PARTNER 3*^17^ (Mean STS 1.9%)	2021	N = 950 496 TAVI 454 SAVR	BE SAPIEN 3 (Edwards)	73	69	99% TAVI 93.8% SAVR	*At 2 years, TAVI vs SAVR: • All-cause death, stroke, or rehospitalization (11.5% vs 17.4%, ss) • Death or Disabling stroke (3% vs 3.8%, ns) • Rehospitalization (8.5% vs 12.5%, ss) • Valve thrombosis (VARC-2) (2.6% vs 0.7%, ss) • Mild PVR (26% vs 2.3%, ss)

	Evolut Low risk trial**^18^ (Mean STS 1.9%)	2023	N = 1414 730 TAVI 684 SAVR	SE CoreValve (3.6%) Evolut R (74.1%) Evolut PRO (22.3%) (Medtronic)	74	65	97.3% TAVI 92.3% SAVR	**At 3 years, TAVI vs SAVR: • All-cause death or disabling stroke (7.4% vs 10.4%, ns) • All-cause death (3.5% vs 4.4%, ns) • Disabling stroke (1.5% vs 2.7%, ns) • Mild PVR (21.3% vs 2.7%, ss) • New PPI (23.2% vs 9.1%, ss)

	NOTION^19^ (Mean STS 3%)	2021	N = 280 145 TAVI 135 SAVR	SE first-generation CoreValve (Medtronic)	79	53	98.5% TAVI 99.3% SAVR	At 8 years, TAVI vs SAVR: • All-cause mortality (51.8% vs 52.6%, ns) • CV death (40.6% vs 43.6%, ns) • Stroke (8.3% vs 9.1%, ns) • New-onset Atrial Fibrillation (50% vs 74.1%, ss) • New PPI (42.5% vs 10.9%, ss)


STS: Society of Thoracic Surgeons; BE: balloon-expandable; SE: self-expandable; TAVI: transcatheter aortic valve replacement; SAVR: surgical aortic valve replacement; CV: cardiovascular; PPI: permanent pacemaker implantation; PVR: paravalvular regurgitation; SVD: structural valve degeneration; AV: aortic valve; ss: statistically significant; ns: statistically nonsignificant * Only 2-year outcomes are available till date. ** 3-year outcomes are available till date.

### Prohibitive-Risk

In 2011, the US Food and Drug Administration (FDA) approved the first-generation balloon-expandable SAPIEN valve (Edwards Lifesciences) in severely symptomatic inoperable aortic stenosis patients after a series of PARTNER trials showed a clinical superiority of TAVI compared with conservative therapy alone.^6,7^ The 5-year follow-up data from PARTNER 1B^8^ continued to demonstrate this superiority. The median survival was 29.7 months post-TAVI versus 11.1 months in the conservative therapy arm. However, it is sobering to note that despite TAVI, overall noncardiac mortality in this population was higher than cardiac mortality (34% vs 17%), highlighting the competing risks of other illnesses and thus the importance of appropriate patient selection for TAVI. Echocardiography showed durable hemodynamic benefit (aortic valve area 1.52 cm² at 5 years, mean gradient 10.6 mm Hg at 5 years) with the rare occurrence of structural valve degeneration (SVD) after TAVI.

Similar 5-year clinical, echocardiographic, and health status outcomes data were observed with the self-expandable supra-annular CoreValve^TM^ (Medtronic Vascular) TAVI,^9^ reassuring 28% survival at 5 years with at least a moderately large quality of life improvement compared to pre-TAVI. The mean transvalvular gradient remained in single digits after TAVI.

### High-Surgical Risk

The 5-year follow-up from the PARTNER 1A study^10^ for high-risk patients showed no difference in the primary outcome of all-cause mortality between SAVR and TAVI (62.4% vs 67.8%, *P* = .76). Functional outcomes were also similar, and preservation of valve hemodynamics was equivalent in both groups. Moderate/severe paravalvular regurgitation (PVR) was significantly higher in the TAVI arm compared with SAVR at 5 years (14% vs 1%, *P* < .001), and even mild PVR portended increased mortality at 5 years. Of note, this study used the first-generation SAPIEN valve, which is now supplanted by newer generation SAPIEN valves.

Gleason et al.^11^ reported analogous 5-year all-cause mortality in high-risk patients with early-generation CoreValve compared to SAVR in the CoreValve US Pivotal High-Risk Trial (55.3% vs 55.4%). More TAVI than SAVR patients underwent reinterventions (3.0% vs 1.1%; *P* = .04) primarily driven by early moderate/severe PVR and had a higher permanent pacemaker placement rate (33.0% vs 19.8%; *P* < .001) by 5 years post-procedure. However, TAVI had a better vascular complication rate profile compared with PARTNER 1A (due to the use of 18F sheaths for CoreValve vs 22F and 24F sheaths in PARTNER).

### Intermediate Surgical Risk

The PARTNER 2 and SURTAVI critical studies compared TAVI with SAVR in intermediate-risk patients with symptomatic severe aortic stenosis using the BE SAPIEN XT valve and SE CoreValve, respectively.^12,13^ At 5 years, primary end points of all-cause mortality or disabling stroke were comparable between transfemoral TAVI to surgery in PARTNER 2.^14^ In contrast to observations in the trials of high-risk patients, cardiac mortality was 56% in each of the intermediate-risk trials.^12,13^ This observation emphasizes the decreasing risk associated with comorbidities that occurs as overall risk decreases. Importantly, the TAVI group had more moderate/severe PVR, which translated into higher 2- to 5-year mortality post-TAVI in landmark analyses.^14^ However, results from a propensity-matched analysis with a new-generation SAPIEN 3 valve suggested a much lower incidence of moderate/severe PVR compared with SAPIEN XT in PARTNER-2 (0.7% vs 6.5%), likely due to the external fabric skirt of SAPIEN 3 valve specifically designed to reduce the incidence of PVR.^15^ Freedom from valve reintervention was better for SAVR compared with TAVI (99.4% vs 96.8%, *P* = .003) at 5 years. The most common reason for reintervention in the TAVI arm was SVD (progressive stenosis or regurgitation). Quality-of-life indices were also similar.

Similar findings were observed in a 5-year follow-up study after SE CoreValve from SURTAVI.^16^ PVR rates were higher with TAVI (3% vs 0.7%, *P* < .001). The mean gradient (8.6 vs 11.2 mm Hg) and effective orifice area (2.2 vs 1.8 cm^2^) were better with TAVI. There were more valve reinterventions post-TAVI (3.5% vs 1.9%, *P* = .02), but the majority were performed within the first 2 years.

### Low Surgical Risk

In 2019, the US FDA approved TAVI in symptomatic elderly patients with severe aortic stenosis and low surgical risk based on the results of two RCTs—PARTNER 3 (using BE SAPIEN 3 valve) and Evolut Low-Risk (using SE CoreValve Evolut).^1,2^

Their long-term results are awaited. However, the 2-year follow-up from the PARTNER 3^17^ trial showed continued superiority of TAVI versus surgery at preventing all-cause mortality, stroke, or rehospitalization but with more frequent deaths, strokes, and valve thrombosis (2.6% vs 0.7%) events between 1 and 2 years. Disease-specific health status at 2 years was better after TAVI than surgery.

Echocardiographic findings through 2 years indicated stable valve hemodynamics and no differences in valve durability parameters. The 3-year follow-up data from the Evolut Low-Risk Trial^18^ showed TAVI to be non-inferior to surgery for the primary end point of all-cause mortality or disabling stroke. Unlike the observations from PARTNER 3, between years 1 and 3 there was no convergence of the primary outcome curves with TAVI. TAVI was associated with superior hemodynamics and significantly less prosthesis-patient mismatch compared with SAVR. However, the incidence of mild PVR (20.3% TAVI vs 2.5% surgery) and permanent pacemaker placement (23.2% TAVI vs 9.1% surgery; *P* < .001) were lower in the surgery group.

## Long-term Outcomes of TAVI

Data from RCTs showing the long-term (> 5 years) outcomes after TAVI compared with SAVR are scarce. Long-term follow-up from the high- and intermediate-risk trials is limited due to shorter patient survival. Younger patients, as enrolled in the low surgical risk trials (mean age 74), constitute the most important group in whom to study long-term TAVI outcomes as well as valve durability.

Currently, the only RCT with more than 5 years of follow-up data available is the Nordic Aortic Valve Intervention (NOTION) trial.^19^ In this all-comers trial, 280 low-risk patients with a mean age of 79 years were randomized to TAVI or SAVR between 2010 and 2013. At 8-year follow-up, the composite outcome and its components—all-cause mortality (51.8% vs 52.6%, *P* = .90), stroke (8.3% vs 9.1%, *P* = .90), and myocardial infarction (6.2% vs 3.8%, *P* = .33)—were similar after TAVI and SAVR. More than mild PVR after TAVI was higher in the NOTION trial than in contemporary practice, often attributed to aortic annulus sizing performed by echocardiography rather than computed tomography and the use of primarily first-generation transcatheter heart valves (THV) without an outer sealing skirt or the possibility of repositioning. The presence of PVR was not associated with an increased risk of death after 8 years of follow-up.

## Significance of THV Durability

With a growing body of literature showing clinical non-inferiority or superiority of TAVI to SAVR in elderly, younger (< 65 years), lower-risk patients often prefer this nonsurgical alternative with the potential for faster recovery with a shorter hospital length of stay. However, SVD is inversely related to age at implantation,^20^ with higher SVD rates of 1.59% per year in younger patients compared with 0.60% per year in middle-aged and elderly patients.^21,22^ The underlying mechanism of the age-related nature of SVD is not fully elucidated. The heightened immune response to the xenograft, increased calcification, and post-implant hemodynamics are some of the proposed mechanisms that cause young patients to demonstrate such aggressive destruction of bioprosthetic heart valves.^21,23,24^ Alternatively, longer survival in younger patients may also increase the likelihood of observing SVD. With a median expected survival of 13 years in 65- to 69-year-old patients undergoing isolated aortic valve replacement,^25^ another valve procedure is very likely if TAVI were performed in this age group. This emphasizes the greater-than-ever need for reliable and comparable data on the long-term durability of surgical and transcatheter bioprostheses before the shared decision-making between patients and the heart team takes place.

## Unique Mechanisms of THV Degeneration

All bioprosthetic valves eventually degenerate. SVD is an inevitable multifactorial process influenced by the patient-, prosthesis- and procedure-related risk factors. As THVs are tissue valves, we may expect similar patient-related (young age, female, diabetes, etc.) and prosthesis-related (stenosis and calcification for bovine valves, leaflet tears and insufficiency for porcine valves, etc.) mechanisms for SVD for THVs.^26^

However, several differences in the preparation and delivery of THVs place them hypothetically at high risk of degeneration.^27^ In contrast to SAVR, during TAVI, THVs are implanted in the native degenerated valves pushing native leaflets apart, ensuring that THVs become embedded inside the bulky calcified annulus. This bulk of calcium along with the prosthetic valve generates turbulence at the aortic root at the sinus of Valsalva, creating chronic mechanical stress on the prosthetic valve leaflets.^28^ Furthermore, the potential leaflet damage during the crimping, loading of THV, asymmetric expansion with suboptimal leaflet coaptation, incomplete frame expansion leading to leaflet-frame interaction, and balloon post-dilation may all accelerate SVD.^26,29^ Finally, the occurrence of subclinical valve thrombosis or hypoattenuated leaflet thickening (HALT) early after TAVI has been detected by 4D multidetector-CT in 10% to 15% of patients and is associated with elevated transprosthetic gradients.^30,31 ^HALT was more frequent in the TAVI arm in the PARTNER 3 trial at 30 days (13% vs 5%, *P* = .03) but not at 1 year (28% vs 20%, *P* = .19).^32^ Spontaneous resolution of 30-day HALT occurred in 54% of patients at 1 year. Patients with HALT at both 30 days and 1 year had significantly increased gradients at 1 year. The Evolut low-risk trial demonstrated similar HALT after TAVI or SAVR at 30 days (17.3% vs 16.5%) and 1 year (30.9% vs 28.4%). Valve gradients were not affected by presence and severity of HALT.^33^ At this time there is no clear evidence that HALT is associated with structural valve degeneration; however, there are signals that patients with HALT have higher mean gradients and inferior hemodynamics in some studies.^30,31,32^ Further data are required on the association or the lack thereof between HALT and structural valve degeneration.

## Caveats of SAVR Durability Assessment

Several studies have shown the encouraging performance of surgical bioprostheses in the first-decade post-surgery with freedom from SVD in > 85% at 10 years.^26,34,35,36,37^ Recent data from the SWEDEHEART registry reported a median survival of 16.2 years (ages 60-64 years) to 6.1 years (age > 85 years) in low-risk patients undergoing bioprosthetic SAVR for severe aortic stenosis.^37^ Historically, structural valve degeneration was defined using clinical end points such as valve reintervention or death related to structural valve failure rather than long-term echocardiographic and hemodynamic assessment. This restricted definition underestimates the true incidence of structural valve degeneration, potentially ignoring a substantial proportion of patients with severe structural valve degeneration who may not undergo reintervention due to advanced age and frailty or early death due to comorbidities even before the onset of structural valve degeneration.^3,26,38^ A systematic review of the surgical literature described that SAVR durability data beyond 10 years originated from a pool of heterogenous studies that used a total of 11 structural valve degeneration definitions in 167 studies.^39^ For instance, Forcillo^40^ reported a 67% freedom from valve reoperation after SAVR, whereas Bourguignon^41^ found freedom from structural valve degeneration as low as 37% using an echocardiography-based structural valve degeneration definition in a 20-year experience with the Carpentier-Edwards Perimount pericardial bioprostheses. Furthermore, studies that reported long-term follow-up (> 20 years) with different surgical valves had wide variability in their real median follow-up, with merely 0.01% to 3% of patients achieving complete 20-year follow-up.^40,41,42,43,44^ It is also noteworthy that the timing of SVD, and thus durability, varies with the type of surgical prosthesis used. Valves such as Sorin Mitroflow (Sorin Group USA) and St. Jude Trifecta experienced structural valve degeneration and failure as early as 4 to 6 years in several studies^40,45,46^ due to the absence of anticalcification treatment during tissue preparation and fixation,^45^ and the early pannus formation and leaflet calcification respectively.^46^ Consequently, wide discrepancies in the structural valve degeneration definitions, incomplete follow-up data, and non-adjusted SVD rates for competing risks such as death make the existing SAVR durability data inadequate to be considered a benchmark for applying catheter-based techniques to younger patients. Therefore, the special emphasis on the randomized data using standardized definitions, long-term core laboratory analysis, and complete follow-up with a specified duration of reporting are paramount.

## Standardized Definition of Bioprosthetic Valve Dysfunction

To streamline the future comparison of long-term durability studies, more sensitive definitions of structural valve degeneration have been proposed by the European Association of Percutaneous Cardiovascular Interventions and the Valve In Valve International Data.^47,48^ Recently, the VARC-3 writing committee^49^ refined this definition. Accordingly, bioprosthetic valve dysfunction may have four underlying pathophysiological mechanisms: (1) structural valve degeneration, which implies irreversible intrinsic changes to structural elements of the valve itself; (2) nonstructural valve dysfunction, which includes PVR and PPM; (3) endocarditis; or (4) thrombosis. The stages of structural valve degeneration are described as Stage 1: morphological valve deterioration without any hemodynamic valve deterioration; Stage 2: moderate hemodynamic valve deterioration; and Stage 3: severe hemodynamic valve deterioration. This revision mandated the presence of permanent structural changes to the prosthetic valve leaflets and the occurrence of hemodynamic valve deterioration during follow-up to confirm the presence of SVD and to differentiate with high residual gradient (≥ 20 mm Hg) related to prosthesis-patient mismatch.^47,50^ An important addition to VARC-3 criteria was the clinical end point of bioprosthetic valve failure, which takes into account the relevant clinical consequences such as Stage 3 SVD-related hemodynamic valve deterioration or irreversible changes in hemodynamics, valve-related death, or valve reintervention.^49^ Using such a universal definition in a contemporary SAVR series, the 10-year rate of ≥ Stage 2 structural valve degeneration was 41%, whereas the rate of structural valve degeneration-related bioprosthetic valve failure was much lower at 3.5%.^51^ Moreover, the occurrence of ≥ Stage 2 structural valve degeneration following SAVR was independently associated with a marked increase in the risk of mortality and valve reintervention during subsequent follow-up.^51,52^

### Death as a Competing Risk

Death is a competing risk factor in long-term follow-up and durability assessment for THVs among high-risk patients with advanced mean age in initial studies. To overcome this limitation, the European Association of Percutaneous Cardiovascular Interventions and VARC-3 recommended using a cumulative incidence function (actual method) in contrast to the Kaplan–Meier analysis (actuarial method).^47,49^ The former methods provide more accurate estimates than the actuarial Kaplan–Meier analysis and may have better clinical utility in the context of TAVI durability studies.

## Long-term Durability of TAVI

Several limitations should be considered when discussing the durability of TAVI.^26^ First, TAVI is a relatively young technology approved nearly a decade ago, precluding long-term durability data to date. Second, current data are available from earlier studies with first-generation THVs that were implanted by relatively inexperienced operators with higher rates of malpositioning and valve sizing issues. Finally, patients in the initial studies included elderly high-risk populations with death as a competing risk factor against the risk of structural valve degeneration over time, limiting the availability of patients for long-term follow-up, as discussed above.^26^

**Figure 1:**
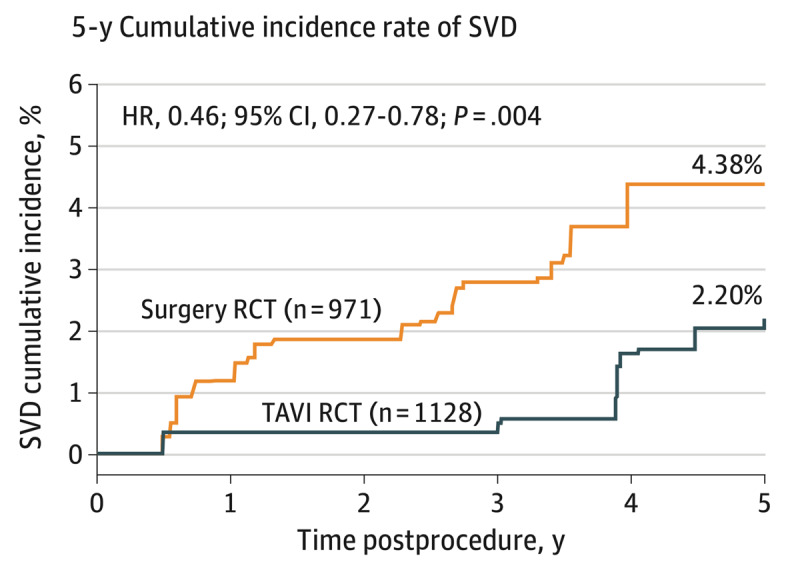
Five-year cumulative incidence rate of structural valve degeneration (SVD) in patients randomized to surgery or transcatheter aortic valve replacement (TAVI) from post hoc analysis pooled data from the CoreValve US High Risk Pivotal (n = 615) and SURTAVI (n = 1484) randomized clinical trials (RCTs). Reproduced with permission from JAMA Cardiol. Copyright©2023 American Medical Association. All rights reserved.^58^

Several registry studies have reported 5 to 7 years of cumulative incidence of structural valve degeneration (using a standardized definition) between 4.8% and 13.3% post-TAVI.^53,54,55,56,57^ Of note, data from RCTs comparing the head-to-head durability of TAVI to SAVR based on new structural valve degeneration definitions is limited (Table 3). THV durability remains difficult to ascertain from the extreme- and high-risk TAVI trials, with a mean age of > 80 years and low survival (28% in extreme risk, < 50% in high risk) at 5 years follow-up. In the CoreValve-High Risk trial,^11^ SVD was defined using EAPCI definitions. The incidence of severe structural valve degeneration was rare and similar between TAVI and SAVR groups. Moderate structural valve degeneration was more common in the SAVR versus the TAVI patients (26.6% vs 9.2%; *P* < .001), although much of this difference was attributable to PPM rather than true SVD. In intermediate-risk patients from the PARTNER 2A trial and SAPIEN 3 registry, Pibarot et al.^50^ found that compared with SAVR, the SAPIEN-XT TAVI cohort had significantly higher 5-year incidence rates of SVD (2.6-fold higher), structural valve degeneration-related bioprosthetic valve failure (4.7-fold higher), and all-cause (structural or nonstructural) bioprosthetic valve failure (*P* ≤ .01), whereas all these rates in the third-generation SAPIEN 3 group were not significant compared with a propensity-score matched SAVR cohort. The majority of bioprosthetic valve failures and valve reinterventions were related to structural valve degeneration in the SAPIEN XT group, PVR in the SAPIEN 3 group, and endocarditis in the SAVR cohort. With a mean age of 81 years in intermediate-risk trials and mortality of 30% to 45% at 5 years, again THV durability remains difficult to ascertain in this patient population. Data on valve durability from the NOTION trial^19^ showed that the risk of SVD (based on imaging data) was lower after TAVI compared to SAVR (13.9% vs 28.3%, *P* = .0017). However, there was no significant difference in the risk of bioprosthetic valve failure, defined as death due to valve dysfunction, hemodynamically significant severe valve dysfunction or aortic valve reintervention between the two groups (8.7% vs 10.5%, *P* = .61). It is notable that despite the NOTION data, including low-surgical-risk patients, the mean age was higher than the pivotal low-risk TAVI trials (mean age 79 vs 74 years) with very few patients in their 60s. In a post hoc analysis pooled data from the CoreValve US Pivotal and SURTAVI trials (n = 4,762, mean age 82.1 yrs), O’hair et al.^58^ reported that a cumulative incidence of SVD, determined by treating death as a competing risk, was lower in patients undergoing TAVI than surgery (2.20% vs 4.38%; HR 0.46; 95% CI, 0.27-0.78; *P* = .004) (Figure 1). This lower risk was most pronounced in patients with smaller annuli (≤ 23 mm diameter; TAVI 1.32%; surgery 5.84%; HR, 0.21; 95% CI, 0.06-0.73; *P* = .02). SVD was also associated with a 5-year increased risk (~2-fold) of all-cause mortality, cardiovascular mortality, and valve disease or worsening heart failure hospitalizations. In summary, current data from these studies confirm that the durability of THVs is comparable to or may be superior to SAVR bioprostheses up to 5 years.

**Table 3:** RCTs comparing long-term durability of TAVI to SAVR using standardized definitions.


STUDIES	SURGICAL RISK	NO. OFPATIENTS	THV (N)SAVR (N)	FOLLOW-UP (Y)	DURABILITYDEFINITION	SVD (%)TAVI VS SAVR, *P*	BVF (%)TAVI VS SAVR, *P*

Pooled analysis of CoreValve US Pivotal and SURTAVI^55^	Inter-mediate/High	4762	CoreValve 3661 Evolut R 130 SAVR 971	5	VARC-3, European task force (2017)	2.2% vs 4.38%, ss	—

PARTNER 2A trial and Sapien 3 registry^47^	Inter-mediate	2329	Sapien XT (774) S3 (891) SAVR (664)	5	VARC-3, European task force (2017)	9.5% Sapien XT vs 3.5%, ss 3.9% S3 vs 3.5%, ns	4.7% Sapien XT vs 1.3%, ss 2.6% S3 vs 1.3%, ns

CoreValve US Pivotal High risk Trial^11^	High	797	CoreValve (391) SAVR (359)	5	European task force (2017)	9.5% vs 26.6%, ss	—

NOTION trial^19^	Low	280	CoreValve (139) SAVR (135)	8	European task force (2017)	13.9% vs 28.3%, ss	8.7% vs 10.5%, ns


RCTs: randomized controlled trials; SVD: structural valve degeneration; BVF: bioprosthetic valve failure; VARC: Valve Academic Research Consortium; P2A: PARTNER 2A; S3: Sapien 3; ss: statistically significant; ns: statistically nonsignificant

### Implications of Aortic Stenosis Lifetime Strategy in the Young

When approaching young patients with aortic stenosis whose life expectancy exceeds the anticipated durability of a valve prosthesis, with more than one reintervention anticipated during their lifetime, the heart team should discuss the impact of the index intervention on future therapeutic options. Instead of generalizing one strategy for all, a patient-tailored approach should be pursued, including choosing the projected sequence of procedures between TAVI and SAVR based on the predicted aspects of the patient (life expectancy, comorbidities), anatomy (aortic root size, height of coronary ostia), the presence of coronary artery disease, anticipated valve characteristics (SVD and durability), technical considerations (need for TAVI explant, risk of coronary obstruction on redo-TAVI) and the operator experience.^59^

## Conclusions

With expanding utilization of TAVI to intermediate- and low-risk young populations with severe AS and longer life expectancy, long-term outcomes and durability data of TAVI bioprostheses are eagerly awaited. This review narrates the promising long-term clinical and functional outcomes of pivotal TAVI RCTs across all risk profiles. As the average complexity of the TAVI patient decreases, the clinical and procedural complications are likely to recede with newer iterations of THVs. Also, the available literature for durability and SVD thus far confirms that THVs are not only comparable to but may also outperform their surgical counterparts at a mid-term follow-up of up to 5 years. However, that finding may or may not be sustained at the long-term follow-up beyond a decade post-TAVI. Therefore, the 10-year follow-up of the low-risk randomized trials (Evolut Low Risk and PARTNER 3) will provide more conclusive data regarding the durability of THVs compared with surgical bioprostheses. Future studies should adhere to the standardized definitions of SVD and bioprosthetic valve failure when reporting valve-related outcomes.

## Key Points

- Transcatheter aortic valve implantation (TAVI) is an established treatment for elderly patients with severe symptomatic aortic stenosis across all surgical risk profiles.
- Growing evidence from the landmark TAVI trials confirms the comparable mid- to long-term (≥ 5 years) clinical and functional outcomes across all risk categories in elderly patients.
- With the expanded use of TAVI in young and low-risk cohorts, it is critical to assess the long-term durability of TAVI before its unrestricted use and before engaging in shared decision-making.
- The use of standardized definitions of structural valve dysfunction should be encouraged to compare the long-term performance of TAVI and surgical aortic valve repair bioprostheses using morphological and hemodynamic parameters.
- TAVI long-term durability data is limited, yet the existing data from randomized clinical trials endorses non-inferior and, in the case of self-expandable transcatheter heart valves, superior durability compared with the surgical bioprostheses at mid-term follow-up.

## CME Credit Opportunity

Houston Methodist is accredited by the Accreditation Council for Continuing Medical Education (ACCME) to provide continuing medical education for physicians.

Houston Methodist designates this Journal-based CME activity for a maximum of 1 AMA PRA Category 1 Credit™. Physicians should claim only the credit commensurate with the extent of their participation in the activity.

Click to earn CME credit: learn.houstonmethodist.org/MDCVJ-19.3.
